# High-flow nasal cannula application in an infant patient with laryngomalacia during general anesthesia: A case report

**DOI:** 10.1097/MD.0000000000028102

**Published:** 2021-12-10

**Authors:** Ji-Yoon Kim, Jieun Bae, Kwang Hyun Lee, Leekyeong Kang, Kyu Nam Kim, Mi Ae Jeong

**Affiliations:** Department of Anesthesiology and Pain Medicine, Hanyang University Medical Center, Hanyang University College of Medicine, Seoul, Republic of Korea.

**Keywords:** high-flow nasal cannula, laryngomalacia, preoxygenation

## Abstract

**Rationale:**

Laryngomalacia is defined as the collapse of supraglottic structures and can cause not only strider but also trigger difficulties with ventilation and endotracheal intubation during anesthesia management. High-flow nasal cannula (HFNC) has been used to manage patients at high risk of hypoxemia in the intensive care unit; however, limited literature information is available for the application of HFNC to infant patients with laryngomalacia during anesthesia practice.

**Patient concerns:**

A 2-month-old male infant was scheduled to undergo surgery for inguinal hernia and undescended testis with general anesthesia.

**Diagnosis:**

The patient had subcostal retraction while breathing and frequent oxygen desaturation events and was diagnosed laryngomalacia.

**Interventions:**

After the patient was supplied oxygen via HFNC and then given general anesthesia, the initial 2 attempts of endotracheal intubation with a rigid laryngoscope were unsuccessful because the vocal cords were obscured by the epiglottis. A third intubation attempt was performed and successful with a 3.0-sized, uncuffed endotracheal tube within 20 minutes of the initial attempt.

**Outcomes:**

No airway complications emerged and oxygen saturation remained at greater than 98% during general anesthesia. The patient was discharged 5 days after surgery without any adverse side effects.

**Lessons:**

Continuous oxygenation via HFNC is a good choice to prevent desaturation during difficult tracheal intubations in infant patients with laryngomalacia. This device is expected to be useful for intubation not only in patients with laryngomalacia, but also in infant patients with a predicted high risk of oxygen desaturation events during general anesthesia.

## 1 Introduction

The use of high-flow nasal cannula (HFNC) has become an increasingly important and popular technique in noninvasive respiratory support to prevent hypoxemia in the intensive care unit (ICU) in the past few years. This therapy was initially introduced in the neonatal ICU as an alternative to noninvasive positive-airway pressure in premature babies^[1]^ and has been used in patients with respiratory distress. Recently, HFNC has come to be considered to have benefits for clinical anesthetic management^[2]^ and pediatric airway surgery.^[3]^

Laryngomalacia is the most common congenital laryngeal disorder in infants, caused by a collapse of the supraglottic structures during inspiration, leading to upper airway obstruction.^[4]^ The symptom like stridor is relieved at the age of approximately 1 year and completely disappear in 24 months in most cases. Because of the laryngomalacia's pathophysiological features make airway management difficult, if the patient requires surgical treatment with general anesthesia, the anesthesiologist should pay attention and closely monitor to prevent hypoxia. Recently, HFNC has been applied during the anesthesia induction and intraoperative period. However, limited data currently support the use of HFNC in infants with laryngomalacia during general anesthesia. Herein, we report a case of successful tracheal intubation and anesthesia management with HFNC in an infant with laryngomalacia.

## 2 Case report

**Figure 1:**
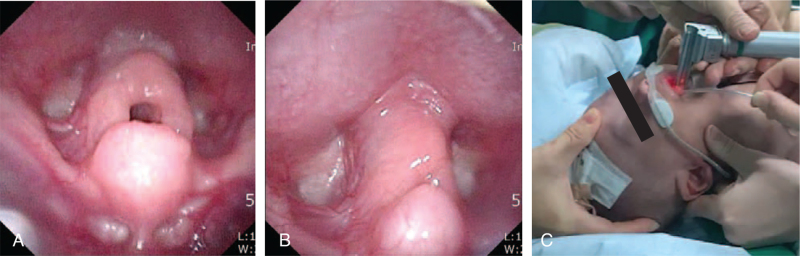
Laryngeal fiberscopic diagnosis of laryngomalacia with abnormal (A) supraglottic edema and omega-shaped epiglottis. (B) Vocal cords were obscured with epiglottis. (C) HFNC application during endotracheal intubation with rigid laryngoscope. HFNC = high flow nasal cannula.

A 2-month-old, 3.9-kg male infant was scheduled to undergo surgery for inguinal hernia and undescended testis with general anesthesia. The baby was delivered 38 + 6 weeks of gestation and a weight of 2440 g via cesarean section. During his hospital stay, the patient had subcostal retraction while breathing and frequent oxygen desaturation events. An otolaryngologist diagnosed laryngomalacia by evaluating the larynx with fiberscopy and finding an omega-shaped epiglottis (Fig. 1A and B). Even after 2 months of observation, the patient displayed laryngomalacia, showing supraglottic edema. However, because the respiratory pattern and the laryngeal edema were improved, an otolaryngologist agreed to pursue surgery with general anesthesia.

Upon arrival to the operating room, the patient was arranged to receive oxygen supply with HFNC (Optiflow System; Fisher and Paekel, Auckland, New Zealand). After vital signs in the normal ranges were confirmed and initial oxygen saturation (SpO_2_) was confirmed at 99%, preoxygenation with 100% oxygen was delivered using a face mask at a rate of 6 L/min for 3 minutes. The patient was then anesthetized with 0.05 mg/kg of midazolam and 0.5 mcg/kg of fentanyl intravenously and, after sedation, 2 L/kg/min of oxygen was delivered via HFNC. The initial 2 attempts of endotracheal intubation with a rigid laryngoscope without a neuromuscular blocking agent (NMBA) were unsuccessful because the vocal cords were obscured by the epiglottis. After deeper sedation was performed with 0.05 mg/kg of midazolam, another intubation attempt was successfully made with a 3.0-sized, uncuffed endotracheal tube within 20 minutes after the initial attempt (Fig. 1C). During intubation, the oxygen saturation was maintained at greater than 98%. NMBA was achieved with 0.6 mg/kg of rocuronium and anesthesia was maintained with sevoflurane in 50% O_2_-air. At the end of the surgery, 0.1 mg/kg of pyridostigmine, and 0.008 mg/kg of glycopyrrolate were administered intravenously to antagonize the residual neuromuscular blockade. After spontaneous breathing had returned, the patient was transferred to the ICU with the endotracheal tube maintained. Dexamethasone (0.2 mg/kg) was injected to prevent airway edema. The next day, the patient showed no desaturation after extubation, although he had chest retraction. The patient was discharged 5 days after surgery without any adverse side effects.

## 3 Discussion

Due to anomalous supraglottic structures, the anesthetic management of infant patients with laryngomalacia relies on an adequate approach to deal with the anticipated difficult airway. In previous reports, spontaneous ventilation was recommended during general anesthesia and sedation was performed without NMBAs. In general, a laryngeal mask airway would be better for general anesthesia than endotracheal intubation for decreasing postoperative coughing.^[5]^ However, in this case, we decided to intubate with an endotracheal tube because the operation time was long and the vocal cords were covered with an omega-shaped epiglottis, which interferes with airway management, including promoting difficulties with ventilation and endotracheal intubation in anesthesia administration.

Regarding airway management, the presence of a difficult airway or delayed intubation is commonly associated with hypoxemia, which can be a life-threatening risk factor for dysrhythmia, brain injury, and even death.^[6]^ Therefore, preoxygenation and rapid endotracheal intubation are needed to prevent hypoxia, especially in some patients who are expected to have a difficult airway, prone to desaturation, or susceptible to apnea, such as pregnant, obese, or pediatric patients or those with laryngomalacia. In pediatric patients, the younger they are, the higher the risk for hypoxia induced by apnea. Conventional oxygen devices like facial masks have been mainly used for oxygenation prior to intubation and should be removed during intubation with the use of a laryngoscope. Therefore, preservation oxygenation during intubation is essential to delay oxygen desaturation. In this case, we attempted continuous oxygenation via HFNC in an infant patient with laryngomalacia during endotracheal intubation, resulting in the maintenance of an oxygen saturation level of greater than 98%.

HFNC, also known as heated, humidified HFNC, is used in the ICU for the management of patients with acute hypoxemic respiratory failure. Therefore, this device facilitates better respiratory mechanics and oxygenation than conventional oxygen devices supplying unheated, dry gas. The delivery of humidified gas also renders some other benefits including improved mucociliary function, thus facilitating secretion clearance, a decreased risk of atelectasis, and an improved ventilation/perfusion ratio and oxygenation. In addition, this type of cannulation reduces some side effects such as mask discomfort, nasal and oral dryness, and eye irritation. Recently, HFNC has increasingly been used during intubation or after extubation to prevent hypoxemia; however, limited cases in anesthesia practice, especially in infants with difficult airways, have been reported. Using HFNC in this case, a continuous oxygen supply was possible during intubation with laryngoscopy and the endotracheal intubation was successful. Moreover, HFNC at a typical dose of 2 L/kg/min retained oxygen saturation at greater than 98% in our infant patient with laryngomalacia. Under these conditions, this case report supports the use of HFNC to perform airway intubation in infant patients with laryngomalacia undergoing surgery.

In conclusion, HFNC is a good choice to prevent desaturation during difficult tracheal intubations in infant patients with laryngomalacia. This device is expected to be useful for intubation not only in patients with laryngomalacia, but also for infant patients with a predicted high risk of oxygen desaturation events during general anesthesia.

## Author contributions

**Conceptualization:** Mi Ae Jeong.

**Data curation:** Ji-Yoon Kim, Jieun Bae, Kwang Hyun Lee.

**Investigation:** LeeKyeong Kang, Kyu Nam Kim.

**Writing – original draft:** Ji-Yoon Kim.

**Writing – review & editing:** Mi Ae Jeong.
